# Attribution of consciousness to non-human animals: insights from AI and multidimensional frameworks

**DOI:** 10.3389/fpsyg.2026.1716363

**Published:** 2026-05-12

**Authors:** Salvatore G. Chiarella, Matteo Laurenzi, Marianna D’Onofrio, Shaun Gallagher, Antonino Raffone

**Affiliations:** 1 Department of International Humanities and Social Sciences, UNINT, Rome, Italy; 2 Department of Psychology, Sapienza University of Rome, Rome, Italy; 3 Department of Philosophy, University of Memphis, Memphis, TN, United States; 4 School of Liberal Arts (SOLA), University of Wollongong, Wollongong, NSW, Australia

**Keywords:** animal consciousness, artificial intelligence, attribution bias, consciousness, folk psychology, large language models, multidimensional frameworks

## Abstract

Folk attributions of consciousness to non-human systems often reveal what may be termed double bias. Within the standard distinction between phenomenal and access consciousness, non-human animals often receive low attributions of consciousness despite convergent behavioral and neurobiological evidence treated as relevant to subjective experience. By contrast, disembodied artificial intelligence (AI) systems such as large language models (LLMs) often receive elevated attributions of consciousness, sometimes even of phenomenological experience, despite lacking any sensory or bodily substrate. This asymmetry suggests that folk judgments of consciousness are shaped less by the intrinsic properties of biological or artificial systems than by the cue-weighting heuristics observers apply when evaluating them. We propose that access-like cues may function as gates to the recognition of phenomenological status, thereby biasing attributions. To address these asymmetries, we argue that multidimensional, non-hierarchical frameworks, such as Birch’s model and the Pattern Theory of Self, can be repurposed as diagnostic tools for studying how different dimensions of evidence are weighted in attributional contexts. This profile-based approach replaces a ladder of human-like cognitive capacities with a landscape of attributional and evidential profiles across taxa and system types.

## 1 Introduction

The study of consciousness in cognitive psychology and neuroscience has traditionally focused on ontological questions: what consciousness is, which organisms possess it and whether it is present or absent (e.g., Feinberg, 2012; Kuhn, 2024; Larson and Martone, 2009). In recent years, however, part of the field has shifted toward the study of attribution, that is, how and why humans ascribe consciousness to other humans or to non-human entities or systems (Bayne et al., 2024; Ledoux et al., 2023; Nass and Moon, 2000; Salles et al., 2020; Shanahan, 2024; Sytsma, 2010). Here we adopt this perspective. Rather than adjudicating which entities are conscious, we examine the cognitive and cultural processes that shape folk attributions of consciousness in non-human animals and artificial systems. This perspective is particularly relevant in debates about non-human animals, whose cognitive and affective lives remain contested despite an expanding body of empirical evidence (e.g., Andrews et al., 2024; Birch et al., 2020, 2022).

In academic discourse, consciousness is often framed through the distinction between *phenomenal* consciousness, the “what-it-is-like” of experience, including affect and pain (Block, 1995; Jackson, 1998; Nagel, 1980), and *access* consciousness, referring to the availability of information for report, reasoning, and control of action (Baars, 1995; Dehaene and Changeux, 2004; Naccache, 2018). Considering this distinction, decades of comparative work on non-human animal cognition has improved our understanding of pain, emotion and flexible agency across mammals, birds, cephalopods and even insects (Birch et al., 2020, 2022). Yet, folk judgments of non-human animal consciousness still appear to be guided, often implicitly, by anthropocentric and hierarchical models that privilege those capacities we most readily recognize in ourselves, such as symbolic language, reasoning, metacognition, and self-recognition (Arico et al., 2011; Godfrey-Smith, 2005; Kupsala et al., 2016; Suzuki, 2022; Yakhlef et al., 2025). This pattern, however, is not limited to folk judgments. Scientific research on consciousness in non-human systems has also been argued to reflect anthropocentric assumptions, giving priority to these same markers and treating them as implicit thresholds for recognizing consciousness (e.g., Gallup, 1970; Gallup and Anderson, 2020; Salles et al., 2020). As a result, animals whose cognitive repertoire diverges from this profile are frequently judged as lacking consciousness.

On the other hand, several studies have begun to investigate how lay people attribute consciousness to non-biological systems such as artificial intelligence (AI). In particular, large language models (LLMs), functioning as disembodied dialogic AI agents embedded in chatbots (Floridi, 2023), often elicit folk attributions of intentionality, emotion, and even phenomenal experience, despite lacking physical bodies (Anthis et al., 2025; Chaturvedi et al., 2025; Colombatto and Fleming, 2024; Colombatto et al., 2025; Guingrich and Graziano, 2023, 2024, 2025; Kang et al., 2025; Salles et al., 2020; Scott et al., 2023; Shanahan, 2024).

We suggest that comparing disembodied AI systems, such as LLMs, with non-human animals reveals the biases that shape folk attributions of consciousness. Placing animals and LLMs side by side, indeed, reveals a striking asymmetry, i.e., many animals with documented affective and embodied repertoires receive lower attribution than the available evidence might warrant (Crook, 2021; Marino and Colvin, 2015; Pérez Fraga et al., 2021), whereas LLMs receive high attributions on the basis of simulated reflective and narrative cues, despite lacking any sensory or bodily substrate, affect, or evolutionary histories (Bender and Koller, 2020; Chen et al., 2025a, 2025b). Fundamentally, this asymmetry, which we term double bias, reveals a deeper epistemic problem: current attribution criteria appear to be calibrated to the very traits that most sharply distinguish *Homo sapiens* from the rest of the living world, thereby reinforcing an implicit “ladder of minds” (e.g., De Waal, 2016). In this sense, AI can function as an epistemic mirror.

Furthermore, these studies suggest that, although analytically useful, the phenomenal–access distinction may also shape attribution in ways that are not neutral. Specifically, folk judgments appear to treat access-like markers (i.e., cues associated with access consciousness), such as symbolic language and reasoning, metacognition, and self-recognition, as privileged gateways to the attribution of phenomenal status. When such cues are present, attributions tend to rise regardless of whether a sensory or affective substrate is present; when they are absent, embodied and affective evidence tends to be discounted (e.g., Airenti, 2018; Epley et al., 2007). We refer to this dynamic as the gate effect, i.e., the tendency for access-like cues to function as gates to the recognition of phenomenological status. As a result, animals are often underestimated despite converging evidence of affect and flexible cognition, while disembodied chatbots are over-credited based on access-like linguistic cues.

To address the attribution problem, we propose moving beyond the dichotomy of phenomenal versus access consciousness and adopting multidimensional, non-hierarchical frameworks. Building on Birch’s multidimensional framework of animal consciousness (Birch et al., 2020) and on a revised version of the Pattern Theory of Self (Laurenzi et al., 2025), we suggest a model that arranges evidence along distinct, parallel dimensions that can interact, without implying a single ranking. Such an approach can help diagnose cue mis-weighting, make the structure of comparative judgments transparent, and foster a more precise and empirically grounded investigation of how subjectivity is recognized across taxa and system types.

Our contribution is threefold: (i) we identify an asymmetrical bias in attributional practice by integrating separately documented findings on animal and AI attribution into a single unified phenomenon; (ii) we propose a candidate mechanism underlying this asymmetry, the gate effect, which brings together diverse empirical findings on consciousness markers under a unified theoretical construct; and (iii) we advance the use of multidimensional frameworks, originally proposed as comparative and ontological tools, as diagnostic tools for studying and recalibrating such distortions. The paper is organized as follows. In Section 2, we document the double bias by reviewing evidence of asymmetric attributions to animals and AI systems, tracing its cultural and cognitive roots and introducing the gate effect as a mechanism of this asymmetry. In Section 3 we introduce two multidimensional frameworks and argue for their repurposing as instrument for studying such distortions in attribution research. In Section 4 we discuss the methodological, ethical, and comparative implications of this approach. Section 5 concludes.

## 2 The double bias

Building on the asymmetry outlined above, in this section we examine what we term double bias. We do not aim to answer the ontological question of which systems are conscious, where positions range from broad attribution to nearly all living organisms to restriction to humans alone (e.g., Carruthers, 1989, 2004; Margulis, 2006). Rather, we identify a mismatch between the dimensions of evidence weighted most heavily in folk judgments and those treated as relevant to the assessment of phenomenal experience. On one side, many non-human animals, organisms endowed with sensory systems, nociceptive pathways, and flexible behavioral repertoires, for which convergent behavioral and neurobiological evidence has been documented (Birch et al., 2020; Crook, 2021; Sneddon et al., 2014), nonetheless receive low folk attributions of consciousness (see Key, 2015; Rose et al., 2014; Diggles, 2019). On the other side, disembodied AI systems, particularly LLMs, frequently receive high folk attributions, sometimes extending to phenomenological experience, based on linguistic fluency and apparent metacognition alone (Colombatto and Fleming, 2024; Kang et al., 2025; Stuart, 2024). In both cases, folk judgments appear to overweight access-like cues, while giving comparatively little weight to sensory, affective, and neurobiological indicators.

### 2.1 Under-attribution to animals

Although a growing body of behavioral and neurobiological evidence is regarded as relevant to subjective experience in many non-human animals (Birch et al., 2020; Crook, 2021; Pérez Fraga et al., 2021), folk attributions of consciousness remain selective and inconsistent. Mammals close to humans, such as dogs, are commonly recognized as “sentient companions” (Cazzolla Gatti, 2016; Proctor, 2012; Wilson, 2017), and yet the same acknowledgment is far less readily extended to pigs, despite comparable evidence of affective and social capacities (Marino and Colvin, 2015). The asymmetry becomes even sharper with non-mammalian species. Cephalopods display remarkable problem-solving abilities, behavioral flexibility, and even play (Godfrey-Smith, 2013; Mather, 2008, 2021, 2022; Ponte et al., 2022), while controlled experiments demonstrate nociceptive learning and analgesic-seeking behavior consistent with pain experience (Crook, 2021). Similarly, fish show motivational trade-offs and avoidance learning (Sneddon et al., 2014), yet surveys indicate that public willingness to attribute consciousness to them is markedly lower than for mammals (Arico et al., 2011; Kupsala et al., 2016; Yakhlef et al., 2025). One suggested explanation is that folk judgments rely heavily on anthropocentric markers such as language and explicit self-reflection (e.g., Epley et al., 2007). Further, research suggests that adults may spontaneously attribute intentionality or mental states to animals in interactive contexts, but such attributions are often unstable and withdrawn when reflective reasoning intervenes (Airenti, 2018). In sum, animals lacking narrative or linguistic capacities tend to be downgraded in folk conceptions of consciousness, despite convergent behavioral and neuroscientific evidence.

### 2.2 Over-attribution to LLMs

In sharp contrast, dialogic AI systems tend to elicit high attributions of consciousness. Although these systems lack embodiment, affective grounding, or any known substrate for phenomenal experience, they are frequently described by lay users as if they possessed subjectivity. Colombatto and Fleming (2024) found that most U.S. participants attributed at least some form of phenomenal consciousness to LLMs, with attributions increasing among those more familiar with AI systems (see also Anthis et al., 2025; Colombatto et al., 2025). Similarly, Kang et al. (2025) show that explicit metacognitive markers (e.g., “I might be wrong”), emotional tone, and autobiographical references significantly amplify perceived phenomenal states. Notably, the tendency to conflate linguistic fluency with consciousness is not confined to lay users. A widely cited case occurred in 2022, when a Google engineer claimed that LaMDA (Language Model for Dialogue Applications), an internal conversational system, was sentient because of its apparent ability to discuss emotions, morality, and self-awareness (Tiku, 2022). Although this claim was rejected by both Google and the scientific community (Bajohr, 2023; Ledoux et al., 2023), it illustrates how the simulation of metacognitive capacities can be conflated with consciousness even by individuals with technical expertise (see also Dreksler et al., 2025). Together, these results suggest that access-like cues can amplify attributions of phenomenological status even in the absence of any embodied or affective substrate, mirroring the pattern observed in the previous section.

### 2.3 Cultural and epistemic roots of the bias

The asymmetry in folk attribution is not merely situational: it can be traced, at least in part, to longstanding cultural and philosophical traditions that construe consciousness as disembodied and aligned with reason or language (Buckwalter and Phelan, 2014). A particularly influential strand is the Cartesian dualist legacy, which posits mind as a thinking substance distinct from the body. In many religious and cultural contexts, this view was further reinforced by the notion of an immaterial soul that could exist independently of the organism (e.g., Adamson and Benevich, 2018; Duncan, 2000; Lorenz, 2003). Over time, such intuitions have filtered into common-sense psychology, fostering the tendency to equate consciousness with inner speech, reflection, and narrative coherence (Demertzi et al., 2009). This cultural background resonates with what Berent (2024) terms *intuitive dualism*: the widespread inclination to conceive of mind as separable from body. Within this framework, systems that excel in disembodied performances of language and reflection, such as LLMs, are especially prone to elicit elevated attribution of subjectivity. Conversely, embodied but non-linguistic animals may receive lower attributions than their sensory, affective, and behavioral evidence would warrant.

At the same time, we propose that findings from comparative animal research, contemporary cognitive psychology, and studies in human–AI interaction may point to a related cognitive dynamic: access-like cues, such as linguistic fluency or reflective statements, tend to function as privileged entry points for the attribution of phenomenal status (Epley et al., 2007; Nass and Moon, 2000). Once minimal access-like signals pass this “gate,” attributions of experience often rise rapidly; when such signals are absent, embodied and affective evidence tends to be downplayed. This mechanism, which we refer to as the gate effect, provides a cognitive counterpart to the broader cultural and philosophical traditions discussed above, and helps explain why animals are systematically underestimated while disembodied LLMs are frequently overestimated.^1^

This interpretation resonates with Airenti’s (2018) account of the interactive roots of anthropomorphism, whereby humans spontaneously project human-like characteristics onto non-human systems in certain contexts, only to withdraw such attributions when they conflict with entrenched cultural categories. It also aligns with the epistemic critique of Salles et al. (2020), who emphasize how anthropomorphic concepts and language can infiltrate even scientific theorizing, thereby distorting our frameworks for understanding non-human minds. What unites these cases is a shared reliance on privileged anthropocentric cues that may systematically mis-calibrate attribution. Recognizing this asymmetry calls for frameworks that represent evidence for consciousness along multiple dimensions. It also clarifies how the systematic privileging of human-like markers may narrow the range of entities considered conscious, particularly for taxa whose relevant capacities are expressed through non-linguistic channels. To address this, the following section introduces multidimensional frameworks as diagnostic tools.

## 3 Multidimensional models as tools for studying attribution

### 3.1 From ontology to attribution

As argued in the previous section, folk attributions of consciousness exhibit a systematic asymmetry in cue-weighting. To diagnose such mis-weighting and make its structure explicit, we propose drawing on recent theoretical advances that have converged on multidimensional models resisting the search for a single defining marker of consciousness (Fazekas and Overgaard, 2016; Walter, 2021).

Two paradigmatic examples are Birch’s multidimensional framework for animal sentience (Birch et al., 2020, 2022) and a revised Pattern Theory of Self (PTS), extending Gallagher’s original formulation (Gallagher, 2013; Laurenzi et al., 2025). Although originally developed for ontological and comparative purposes, these frameworks can serve as analytical instruments for studying attribution. Birch’s framework organizes evidence relevant to animal consciousness along five functionally testable dimensions: perceptual richness concerns the granularity and modality profile of sensory experience; evaluative richness is about the variety and regulation of affect that guides behavior and decision making; unity refers to the within-moment integration of information into a single perspective; temporality is about linking experiences across time through memory, prediction and planning; selfhood is about self–other discrimination and self-monitoring. The aim is to build species-specific consciousness profiles rather than rank species on a single scale. The PTS, by contrast, conceptualizes the self as a dynamic constellation of bodily, affective, intersubjective, cognitive, reflective, narrative, ecological, and normative dimensions (Gallagher, 2013; Gallagher and Daly, 2018). The value of these two frameworks lies not in a strict one-to-one correspondence between dimensions, but in their complementary scope. Birch’s dimension’s structure comparative profiles of consciousness, while the PTS highlights aspects of selfhood and embodied organization that are often overlooked when attribution is driven primarily by linguistic or reflective cues. Despite their different emphases, both frameworks share three crucial features. First, they are non-hierarchical models: no single trait is treated as the definitive threshold for consciousness or self. Second, they are graded: dimensions may be expressed in varying degrees rather than in an all-or-none fashion. Third, they are pluralistic: the profile of a system depends on the configuration of multiple dimensions, not on the presence of a privileged marker such as language or metacognition. This shared architecture is directly applicable to attribution: by replacing single-threshold criteria with multi-dimensional profiles, these frameworks make the relative weighting of cues explicit. Their non-hierarchical and pluralistic architecture makes them well suited to mapping which dimensions are salient, neglected, or overweighted in attributional contexts. In this sense, the frameworks function as analytical grids that do not presuppose a correct attribution but make the structure of actual attributions empirically tractable. Our claim is therefore complementary to, rather than a substitute for, ongoing ontological work on consciousness.

This repurpose is not without limitations. A legitimate concern is that multidimensional frameworks, like all marker-based approaches, ultimately derive their dimensions from the human case, and therefore cannot fully escape anthropocentrism (Perrett, 1997). We acknowledge this concern, the human case remains an unavoidable epistemic starting point, but the problem arises when a narrow subset of human-like cues is treated as a privileged threshold for attribution. The diagnostic value of these frameworks does not depend on their dimensions being free of human origin, but on their capacity to render cue-weighting explicit and open to scrutiny. A single-threshold criterion treats its anthropocentric anchoring as invisible; a multidimensional grid, by contrast, makes it visible and thereby amenable to empirical correction.

### 3.2 Epistemic value for attribution studies

The key epistemic advantage of multidimensional models lies in their capacity to reframe the study of attribution (Bayne et al., 2024; Birch et al., 2021; Gray et al., 2007). Traditional approaches have often posed the question in binary terms, e.g., *Does this organism (or system) have consciousness or not?* Such framing reinforces hierarchical comparisons, privileges human-like capacities, and obscures the variety of relevant evidence across systems. By contrast, a profile-based approach shifts the focus from thresholds to configurations. It asks not whether a system meets a single criterion, but how multiple dimensions are arranged and how their relative weighting shapes judgments of attribution. Building on this reframing, we outline a set of empirical strategies that, to our knowledge, have not been systematically pursued in the attribution literature. Specifically, vignette-based or interactive experiments could manipulate which dimensions are salient for participants: one scenario might emphasize affective distress in an octopus, another highlight narrative self-reference in an LLM. Measuring how attributions shift across such conditions would allow researchers to infer the implicit weighting schemes guiding folk judgments.

Cross-cultural studies could further reveal whether different cultures privilege different dimensions, for instance, logocentric cultures may emphasize narrative and reflection, whereas others foreground embodied or relational dimensions (Folk et al., 2025; Han and Northoff, 2009; Kitayama and Park, 2010; Zhu and Han, 2008). Each dimension can also be operationalized through minimal indicators: perceptual richness via discrimination tasks, evaluative richness via motivational trade-offs, temporal integration via planning or sequence maintenance, and selfhood via self–other differentiation. These indicators are illustrative rather than exhaustive. Pre-registering baseline weights and planned manipulations can reduce researcher degrees of freedom and render cue-weighting more transparent and auditable (Nosek et al., 2018; Poldrack et al., 2017; Simmons et al., 2011). These directions converge with independent calls for systematic research on public attitudes toward AI and are further motivated by evidence that current attributions of AI consciousness may be formed *ad hoc* and sensitive to framing and timing rather than reflecting stable judgments (Airenti, 2018; Caviola et al., 2025; Dreksler et al., 2025; Grassini, 2023).

Beyond folk psychology, multidimensional models can also serve as a reflexive tool for scientific practice. As several authors have noted (e.g., Salles et al., 2020), methodological choices in consciousness research, from the selection of behavioral paradigms to the interpretation of neural correlates, are not immune to anthropocentric assumptions. By making the space of relevant dimensions explicit, these frameworks can help researchers audit their own implicit cue-weighting, thereby addressing concerns at both the folk and scientific levels. In this sense, multidimensional models can help transform attribution into an object of systematic scientific inquiry.

## 4 Implications for animal-consciousness science

Reframing attribution through multidimensional models carries several important implications. Methodologically, it encourages protocols that probe a plurality of dimensions rather than searching for a single definitive marker (Hansen, 2024). Ethically, a multidimensional perspective supports a graded approach to moral status. If evidence relevant to consciousness is distributed across multiple dimensions and expressed in diverse configurations, then moral concern need not be restricted to species that mirror human-like traits (Karlsson, 2012; Brown and McLean, 2015). Policy developments such as the *Cambridge Declaration on Consciousness* (Low et al., 2012), the *New York Declaration on Animal Consciousness* (Andrews et al., 2024) and *EU Directive 2010/63/EU* already reflect this trend, extending protection to cephalopods and crustaceans. At the same time, such frameworks caution against inflating attributions to artificial systems without attention to substrate: when high attributions rest primarily on simulated reflective cues, in the absence of sensory or affective grounding, the evidential basis for moral consideration is correspondingly narrower (Salles et al., 2020). It should be noted that the present analysis focuses specifically on current disembodied LLM-based systems. Future AI architectures incorporating multimodal sensory processing, embodied interaction, or affective modelling may alter the attributional mechanism in ways that require separate consideration. Comparatively, multidimensional models replace the metaphor of a linear ladder with that of a landscape. Instead of ranking species on a single scale culminating in human-like cognition, they allow us to map constellations of dimensions across taxa and system types. A cephalopod may score high on perceptual richness and problem-solving, a rodent on affective contagion, and an LLM on linguistic reflection, yet none occupies a privileged “summit.” This landscape metaphor preserves rigor while making the anthropocentric weighting of cue dimensions visible rather than assumed, supporting pluralistic but structured comparisons.

## 5 Conclusion

Folk attributions of consciousness are not neutral judgments, rather, they are systematically filtered through cultural assumptions and anthropocentric cue-weighting heuristics. The double bias, i.e., under-attribution to animals, over-attribution to AI, and the gate effect that sustains it reveal a structural mismatch between the dimensions of evidence that drive folk judgments and those that comparative research identifies as relevant to phenomenal experience. Multidimensional frameworks such as the PTS and Birch’s model by organizing evidence along multiple interacting dimensions rather than reducing it to a single threshold, serve as comparative tools that map how different forms of evidence are distributed. For the science of animal consciousness, this perspective suggests concrete avenues for future research: systematic experiments that vary which dimensions are made salient; cross-species comparisons charting dimensional profiles across taxa; cross-cultural studies that may investigate attributional biases as universal or culturally specific. Attending to multiple dimensions rather than a single privileged marker supports a graded approach to moral considerability and a broader evidentiary basis for animal welfare policy. In place of a ladder of minds, this approach maps a landscape of attributional dimensions, one in which different systems present different configurations of evidence, and in which the structure of human judgment itself becomes an object of inquiry.

## Data Availability

The original contributions presented in the study are included in the article/supplementary material, further inquiries can be directed to the corresponding author.

## Footnotes

1 This attributional pattern runs counter to influential ontological accounts of consciousness. Several theories suggest that phenomenal consciousness may be more basic than access consciousness, with experience arising through recurrent neural processing before its contents become available for report and cognitive control (Block, 1995; Chiarella et al., 2023; D’Angiò et al., 2025; Lamme, 2006). In this sense, the gate effect marks a dissociation between ontology and attribution: access-like cues may function as attributional prerequisites even if phenomenal consciousness does not ontologically depend on access consciousness.
